# Long-term increase in fasting blood glucose is associated with increased risk of sudden cardiac arrest

**DOI:** 10.1186/s12933-023-01764-0

**Published:** 2023-02-20

**Authors:** Yun Gi Kim, Seung-Young Roh, Joo Hee Jeong, Hyoung Seok Lee, Kyongjin Min, Yun Young Choi, Kyung-Do Han, Jaemin Shim, Jong-Il Choi, Young-Hoon Kim

**Affiliations:** 1 Division of Cardiology, Department of Internal Medicine, Korea University College of Medicine and Korea University Anam Hospital, 73 Goryeodae-Ro, Seongbuk-Gu, Seoul, 02841 Republic of Korea; 2 Division of Cardiology, Department of Internal Medicine, Korea University College of Medicine and Korea University Guro Hospital, Seoul, Republic of Korea; 3 Department of Statistics and Actuarial Science, Soongsil University, Seoul, Republic of Korea

**Keywords:** Sudden cardiac arrest, Diabetes mellitus, Fasting blood glucose

## Abstract

**Background:**

Diabetes mellitus (DM) is associated with various cardiovascular complications, including sudden cardiac arrest (SCA). Furthermore, the severity of DM, as assessed by fasting blood glucose (FBG), is associated with the risk of SCA. However, whether long-term changes in FBG influence on SCA risk remains to be determined.

**Methods:**

This study used sequential nationwide health screening data from 2009 and 2011. FBG was measured at each health screening, and ΔFBG was calculated as FBG in 2011–FBG in 2009.

**Results:**

Overall, 2,801,153 people were analyzed, and the mean follow-up duration was 6.33 years. Compared with the euglycemic group (− 20 ≤ ΔFBG < 20), the 20 ≤ ΔFBG < 40, 40 ≤ ΔFBG < 100, and ΔFBG ≥ 100 groups had increased SCA risks of 25% (adjusted hazard ratio [HR] = 1.25; 95% confidence interval [CI] 1.16–1.35; p < 0.001), 66% (adjusted HR = 1.66; 95% CI 1.49–1.86; p < 0.001), and 2.9-fold (adjusted HR = 2.85; 95% CI 2.37–3.44; p < 0.001), respectively. The association between ΔFBG and SCA was maintained in people with DM but not in people without DM. However, sex, age, blood pressure, and presence of heart failure did not affect the association between ΔFBG and SCA. A decrease in ΔFBG over time was not associated with reduced risk of SCA: the adjusted HR was 1.11 (95% CI 0.98–1.27; p = 0.113) for the ΔFBG < –40 group and 1.12 (95% CI 1.03–1.22; p = 0.009) for the − 40 ≤ ∆FBG < − 20 group.

**Conclusions:**

A long-term increase in ΔFBG can be associated with increased risk of SCA in people with DM. However, a long-term decrease in ΔFBG was not associated with reduced risk of SCA. Actions to prevent increase in FBG can have significant effects on public health in terms of SCA prevention.

**Supplementary Information:**

The online version contains supplementary material available at 10.1186/s12933-023-01764-0.

## Background

Sudden cardiac arrest (SCA) is a major healthcare burden which leads to multi-organ failure and death if left untreated [1–4]. The clinical value of various therapeutic interventions to bring the patient back to life, such as induced hypothermia, antiarrhythmic drugs, and coronary angiography, remains uncertain [5–7]. Although high quality bystander cardiopulmonary resuscitation immediately after SCA accompanied by the use of automatized defibrillators is associated with improved survival of SCA patients [8–10], not all SCA events are witnessed, and automatized defibrillators are not readily available in most places. Furthermore, educating citizens to perform high-quality bystander cardiopulmonary resuscitation is difficult to achieve. Therefore, prevention rather than treatment of SCA deserves more attention.

A prior study demonstrated that the risk of SCA is substantially increased not only in people with diabetes mellitus (DM), but also in people with impaired fasting blood glucose (FBG) [11]. Furthermore, the risk of SCA showed a linear association with FBG, with people having FBG ≥ 200 mg/dL showing the highest risk [11]. However, whether an increase in FBG over time could increase the risk of SCA remains to be determined. The effects of a reduction of serum glucose on SCA also are under debate. The ACCORD and ADVANCE trials of intensive blood glucose lowering therapy showed no benefit in preventing macrovascular complications or delaying death in type 2 DM patients [12, 13]. However, the UKPDS, EMPA-REG OUTCOME, and LEADER trials suggested a potential cardiovascular mortality benefit of metformin, empagliflozin, and liraglutide in DM patients, respectively [14–16]. In this study, we evaluated the association between long-term change in FBG, which can be a surrogate marker for serum glucose control, and the risk of SCA in population data from the Republic of Korea nationwide healthcare insurance system.

## Methods

### Study cohort

This study is a retrospective cohort analysis based on the Korean National Health Insurance Service (K-NHIS) database. All people living in the Republic of Korea are mandatory subscribers of the K-NHIS, the medical insurance system managed by the Korean government. Therefore, the K-NHIS represents the entire population of the Republic of Korea. Upon approval from the official K-NHIS review committee (https://nhiss.nhis.or.kr/), researchers can use the K-NHIS database to perform various medical studies.

As with other claims-based databases, the K-NHIS database stores claims using International Classification of Disease, 10th edition (ICD-10) diagnostic codes and prescriptions for drugs. However, Korea’s regular nationwide health screening program, which is offered biennially to subscribers, is a strong advantage of K-NHIS data over other claims-based databases. Each health screening collects: (i) medical measurements such as systolic and diastolic blood pressure, body weight, height, and waist circumference; (ii) self-report questionnaires about smoking status, alcohol consumption habits, and exercise level; and (iii) laboratory tests such as complete blood cell counts, serum creatinine, liver function, lipid profiles, and FBG. Because all NHIS subscribers are encouraged to participate in the nationwide health screening every two years, researchers can obtain data showing changes in blood pressure, lipid profiles, and FBG over time.

For this study, people who underwent nationwide health screening in both 2009 and 2011 were screened for eligibility. The exclusion criteria were: (i) diagnosis of SCA before the 2011 health check-up, (ii) younger than 20 years, and (iii) prior medical history of respiratory failure; critical bleeding; sepsis; anaphylaxis; trauma; or accidents such as suffocation, lightning strike, drowning, burns, or electrocution within 6 months of the 2009 health check-up. To identify participants’ baseline medical history, such as hypertension, DM, heart failure, atrial fibrillation, and dyslipidemia, data obtained from January 2002 to December 2011 were used. Participants were followed until December 2018.

Both the Institutional Review Board of Korea University Medicine Anam Hospital and the official review committee of K-NHIS approved this study. The requirement for written informed consent was waived due to the retrospective nature of the study. This study strictly followed both the legal regulations of the Republic of Korea and the ethical guidelines of the 2013 Declaration of Helsinki.

### Definitions

Fasting blood glucose was measured in both 2009 and 2011 nationwide health screenings, and ∆FBG was defined as FBG in 2011–FBG in 2009. Baseline demographics were obtained during the 2009 health screening. The study participants were classified into six groups based on ∆FGB (mg/dL): (i) ∆FBG < − 40; (ii) − 40 ≤ ΔFBG < − 20; (iii) − 20 ≤ ΔFBG < 20; (iv) 20 ≤ ΔFBG < 40; (v) 40 ≤ ΔFBG < 100; and (vi) ΔFBG ≥ 100.

Alcohol consumption was defined as: (i) non-drinkers: 0 g per week; (ii) mild- to moderate-drinkers: 0 g to 210 g per week; and (iii) heavy-drinkers: 210 g or more per week. Smoking status was classified as: (i) never-smokers: those who smoked < 100 cigarettes in their lifetime; (ii) ex-smokers: people who smoked more than 100 cigarettes in their lifetime but had not smoked within 1 month of the 2009 health screening; (iii) current-smokers: people who smoked more than 100 cigarettes and continued to smoke within one month of the 2009 health screening. Hypertension was defined as a claim with ICD-10 codes for hypertension or a measured systolic blood pressure (SBP) ≥ 140 mmHg or diastolic blood pressure ≥ 90 mmHg during the 2009 health screening. DM was defined as either a prior diagnosis of DM by a physician (ICD-10 codes for DM) or a measured FBG ≥ 126 mg/dL during the 2009 health check-up. Dyslipidemia was diagnosed as a prior claim with ICD-10 codes for dyslipidemia. The estimated glomerular filtration rate (eGFR) was calculated with the Modification of Diet in Renal Disease equation using the creatinine level measured during 2009 health check-up, and chronic kidney disease (CKD) was defined as an eGFR < 60 mL/min/1.73 m^2^. Regular physical activity was defined as performing one or more high-intensity (such as running, climbing, or intense bicycle activities) or moderate-intensity (such as walking fast, tennis, or moderate bicycle activities) exercise sessions per week. Our prior studies have demonstrated the robustness of these definitions [4, 17–20].

### Primary outcome endpoint

The occurrence of SCA was the main outcome of this study, and both aborted and non-aborted SCAs were included. The diagnosis of SCA was based on ICD-10 codes during an emergency department visit: I46.0 (cardiac arrest with successful resuscitation), I46.1 (sudden cardiac arrest), I46.9 (cardiac arrest, cause unspecified), I49.0 (ventricular fibrillation and flutter), R96.0 (instantaneous death), and R96.1 (death occurring less than 24 h from symptom onset). The incidence of SCA is reported as event number per 1000 person-years of follow-up.

Claims with ICD-10 codes for SCA can arise from new occurrences of SCA or repeat claims of an SCA that occurred previously. If a claim containing ICD-10 codes for SCA occurred immediately after the start of clinical follow-up (after 2011 health screening), we could not discriminate whether it reflected a new occurrence of SCA or a repeat claim for a prior SCA event. To prevent errors originating from repeat claims, claims for SCA or death that occurred within one year after the 2011 health screening (start of clinical follow-up) were not counted as a main outcome.

### Statistical analysis

The Kolmogorov–Smirnov test was used to test the normal distribution of continuous variables. All continuous variables in this study was normally distributed and Student’s *t*-test was used for comparison. Categorical variables were compared with Chi-square test since all expected count was equal or greater than five. Hazard ratios (HRs) and their 95% confidence intervals (CIs) were calculated through a Cox-regression analysis. Age, sex, body mass index (BMI), income, smoking status, alcohol consumption status, regular physical activity, hypertension, dyslipidemia, CKD, heart failure, and baseline FBG (measured in 2009) were adjusted in the multivariable Cox-regression model. All tests were two-tailed, with p values ≤ 0.05 indicating statistical significance. SAS version 9.4 (SAS Institute, Cary, NC, USA) was used for all statistical analyses.

## Results

### Patients

**Fig. 1:**
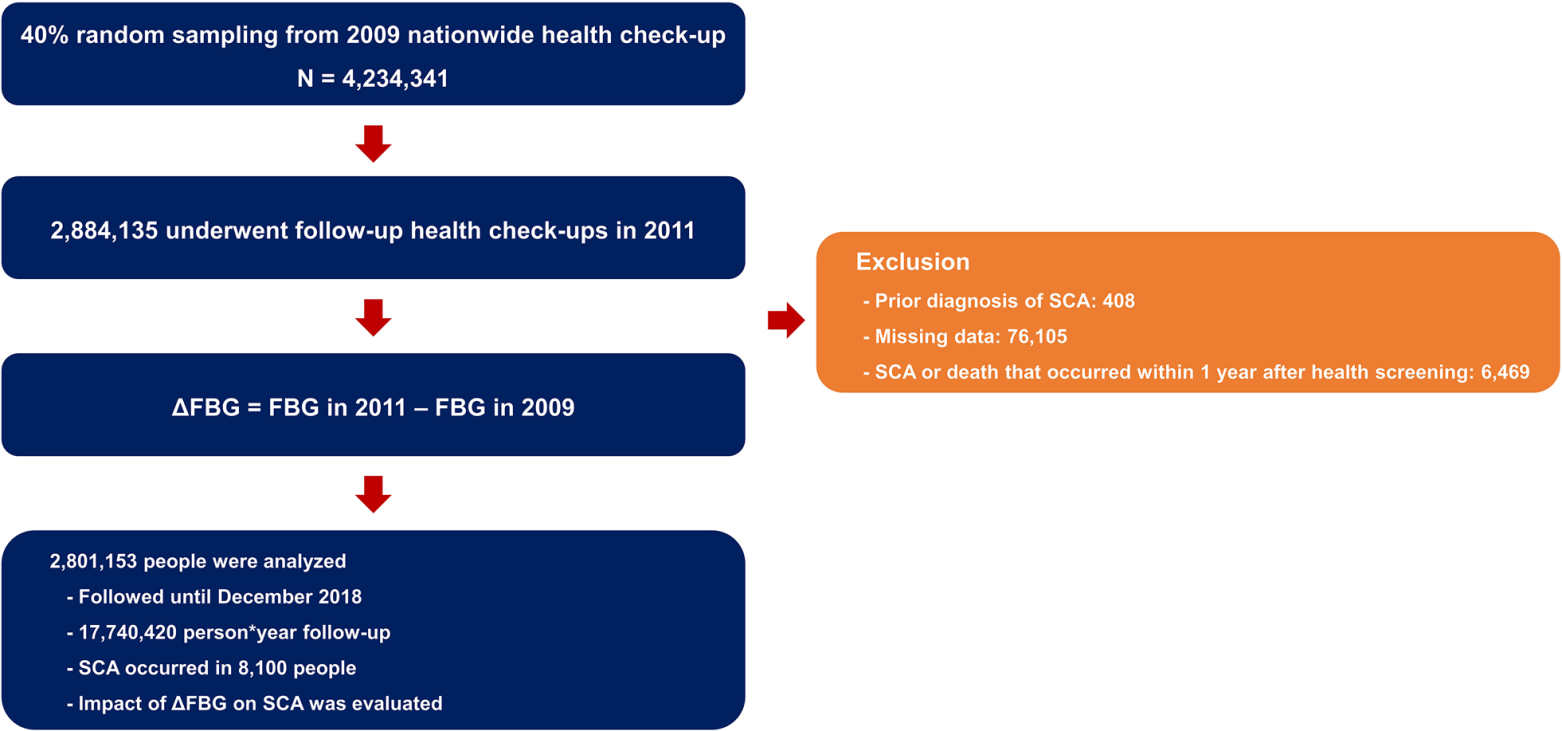
Flow of the study. FBG, fasting blood glucose; SCA, sudden cardiac arrest

We randomly selected 40% of the 10 million people who underwent a nationwide health screening in 2009 (n = 4,234,341). Among them, a total of 2,884,135 people also underwent a health screening in 2011. People with missing data (n = 76,105) or a diagnosis of SCA before the 2009 health check-up (n = 408) and those who had a claim with ICD-10 codes for SCA within one year after the 2011 health screening (n = 6469) were excluded from the analysis, so 2,801,153 people were analyzed. Fasting blood glucose was measured in both the 2009 and 2011 health screenings, and ∆FBG was calculated as FBG in 2011–FBG in 2009 (md/dL). The flow of this study is summarized in Fig. 1.

During a mean follow-up duration of 6.33 years (17,740,420 person*years of follow-up), 8100 SCA events occurred, for an incidence of 0.457 per 1000 person*years. Significant differences according to the presence of an SCA event were observed and described in our previous report [4, 11]. People who experienced an SCA event during follow-up were older; more likely to be male, smokers, and in the lowest quintile of income; and had a higher prevalence of DM, hypertension, dyslipidemia, and CKD. The baseline demographics according to DM status (non-DM vs. impaired fasting blood glucose [IFG] vs. DM) are summarized in Table 1. People with DM were older; more likely to be male and in the lowest quintile group of income; had a higher prevalence of hypertension, dyslipidemia, CKD, atrial fibrillation, and heart failure; and a higher BMI, FBG, and blood pressure. However, people with DM had lower levels of total cholesterol and low-density lipoprotein than the other groups, and that was statistically significant even though the absolute difference was not significant. The percentage of people performing regular exercise was significantly higher among people with DM than in the other groups. Because we intended to evaluate the correlations between ∆FBG and SCA, we also defined the baseline characteristics according to degree of ∆FBG (Table 2). Most people maintained their ∆FBG to within 20 mg/dL (n = 2,346,956, 83.8%). People who showed extreme changes in FGB (∆FBG < − 40 and ∆FBG > 100) were older; more likely to be male, smokers, heavy-drinkers, and in the lowest quintile of income; had higher BMI, blood pressure, and FBG; and had higher prevalence of DM, hypertension, dyslipidemia, CKD, atrial fibrillation, and heart failure. People with ∆FBG < − 40 had a higher percentage of regular exercise and the lowest level of total cholesterol. Baseline FBG were significantly higher in ∆FBG < − 40 group.

**Table 1:** Baseline demographics

	Presence of diabetes mellitus			p value
	Non-diabetic	Impaired fasting glucose	Diabetes mellitus	
	n = 1,902,798	n = 639,231	n = 259,124	
*Male*	1,021,897 (53.7%)	416,920 (65.22%)	162,367 (62.66%)	< 0.001
*Age*	46.85 ± 13.35	51.16 ± 12.82	58.41 ± 11.65	< 0.001
*Age groups*				< 0.001
20–29	173,978 (9.14%)	21,198 (3.32%)	1695 (0.65%)	
30–39	444,623 (23.37%)	103,963 (16.26%)	14,032 (5.42%)	
40–49	501,451 (26.35%)	167,120 (26.14%)	41,570 (16.04%)	
50–59	426,703 (22.43%)	177,510 (27.77%)	76,319 (29.45%)	
60–69	226,360 (11.9%)	105,773 (16.55%)	73,329 (28.3%)	
70–79	114,460 (6.02%)	55,506 (8.68%)	46,410 (17.91%)	
80–	15,223 (0.8%)	8161 (1.28%)	5769 (2.23%)	
*Body mass index*	23.45 ± 3.09	24.48 ± 3.14	25.03 ± 3.28	< 0.001
*Waist circumference*	79.28 ± 8.86	82.78 ± 8.48	85.4 ± 8.41	< 0.001
*Smoking status*				< 0.001
Non-smoker	1,156,003 (60.75%)	336,345 (52.62%)	141,973 (54.79%)	
Ex-smoker	291,112 (15.3%)	134,392 (21.02%)	55,205 (21.3%)	
Current smoker	455,683 (23.95%)	168,494 (26.36%)	61,946 (23.91%)	
*Alcohol consumption*				< 0.001
Non-drinker	979,705 (51.49%)	292,136 (45.7%)	146,764 (56.64%)	
Mild drinker	805,037 (42.31%)	284,774 (44.55%)	89,441 (34.52%)	
Heavy drinker	118,056 (6.2%)	62,321 (9.75%)	22,919 (8.84%)	
*Regular exercise*	373,528 (19.63%)	136,411 (21.34%)	60,359 (23.29%)	< 0.001
*Lowest quintile of income*	314,936 (16.55%)	106,221 (16.62%)	48,896 (18.87%)	< 0.001
*Fasting blood glucose (mg/dL)*	88.07 ± 7.42	107.59 ± 6.47	141.75 ± 44.92	< 0.001
*Hypertension*	403,275 (21.19%)	226,931 (35.5%)	156,046 (60.22%)	< 0.001
*Hypertension groups*				< 0.001
Non-hypertensive	742,754 (39.03%)	148,248 (23.19%)	34,715 (13.4%)	
Pre-hypertension	756,769 (39.77%)	264,052 (41.31%)	68,363 (26.38%)	
Hypertension	120,296 (6.32%)	63,122 (9.87%)	19,364 (7.47%)	
Hypertension with medication	282,979 (14.87%)	163,809 (25.63%)	136,682 (52.75%)	
*Systolic blood pressure (mmHg)*	120.6 ± 14.12	125.79 ± 14.45	128.58 ± 15.23	< 0.001
*Diastolic blood pressure (mmHg)*	75.37 ± 9.64	78.39 ± 9.76	78.58 ± 9.97	< 0.001
*Dyslipidemia*	301,053 (15.82%)	156,489 (24.48%)	117,079 (45.18%)	< 0.001
*Dyslipidemia groups*				< 0.001
Total cholesterol < 240 (mg/dL)	1,601,745 (84.18%)	482,742 (75.52%)	142,045 (54.82%)	
Total cholesterol ≥ 240	137,075 (7.2%)	68,984 (10.79%)	18,501 (7.14%)	
Total cholesterol ≥ 240 with medication	163,978 (8.62%)	87,505 (13.69%)	98,578 (38.04%)	
*Total cholesterol (mg/dL)*	193.37 ± 35.02	201.71 ± 37.41	191.19 ± 41.63	< 0.001
*High-density lipoprotein (mg/dL)*	55.9 ± 18.23	54.58 ± 22.77	51.14 ± 23.36	< 0.001
*Low-density lipoprotein (mg/dL)*	113.91 ± 42.01	119.01 ± 48.74	108.34 ± 46.43	< 0.001
*Chronic kidney disease*	81,552 (4.29%)	37,783 (5.91%)	27,786 (10.72%)	< 0.001
*eGFR (mL/min/1.73m*^*2*^*)*	90.6 ± 38.44	87.24 ± 33.33	85.64 ± 36.91	< 0.001
*Atrial fibrillation*	25,557 (1.34%)	11,564 (1.81%)	8508 (3.28%)	< 0.001
*Heart failure*	35,715 (1.88%)	16,789 (2.63%)	15,209 (5.87%)	< 0.001

eGFR, estimated glomerular filtration rate

**Table 2:** Baseline demographics of patients stratified by ∆FBG

	ΔFBG (mg/dL)						p value
	ΔFBG < − 40	− 40 ≤ ΔFBG < − 20	− 20 ≤ ΔFBG < 20	20 ≤ ΔFBG < 40	40 ≤ ΔFBG < 100	ΔFBG ≥ 100	
	n = 49,891	n = 146,978	n = 2,346,956	n = 200,279	n = 48,344	n = 8705	
*Male*	33,596 (67.34%)	90,647 (61.67%)	1,309,868 (55.81%)	127,859 (63.84%)	32,961 (68.18%)	6253 (71.83%)	< 0.001
*Age*	54.99 ± 12.78	50.9 ± 13.88	48.41 ± 13.46	50.31 ± 13.76	53.83 ± 13.08	54.01 ± 12.43	< 0.001
*Age groups*							< 0.001
20–29	1128 (2.26%)	8522 (5.8%)	174,359 (7.43%)	11,425 (5.7%)	1318 (2.73%)	119 (1.37%)	
30–39	5221 (10.46%)	25,613 (17.43%)	487,119 (20.76%)	37,501 (18.72%)	6169 (12.76%)	995 (11.43%)	
40–49	9920 (19.88%)	33,761 (22.97%)	606,482 (25.84%)	47,663 (23.8%)	10,247 (21.2%)	2068 (23.76%)	
50–59	14,655 (29.37%)	36,520 (24.85%)	562,749 (23.98%)	50,156 (25.04%)	13,852 (28.65%)	2600 (29.87%)	
60–69	11,523 (23.1%)	25,743 (17.51%)	324,407 (13.82%)	32,047 (16%)	9944 (20.57%)	1798 (20.65%)	
70–79	6485 (13%)	14,751 (10.04%)	169,576 (7.23%)	18,622 (9.3%)	5978 (12.37%)	964 (11.07%)	
80–	959 (1.92%)	2068 (1.41%)	22,264 (0.95%)	2865 (1.43%)	836 (1.73%)	161 (1.85%)	
*Body-mass index*	24.47 ± 3.29	23.94 ± 3.18	23.74 ± 3.14	24.35 ± 3.31	24.93 ± 3.49	25.03 ± 3.66	< 0.001
*Waist circumference*	83.94 ± 8.65	81.52 ± 8.86	80.25 ± 8.94	82.49 ± 8.94	84.94 ± 8.87	85.95 ± 8.95	< 0.001
*Smoking status*							< 0.001
Non-smoker	25,136 (50.38%)	79,936 (54.39%)	1,396,268 (59.49%)	105,397 (52.63%)	23,587 (48.79%)	3997 (45.92%)	
Ex-smoker	9860 (19.76%)	26,619 (18.11%)	395,626 (16.86%)	37,394 (18.67%)	9488 (19.63%)	1722 (19.78%)	
Current smoker	14,895 (29.86%)	40,423 (27.5%)	555,062 (23.65%)	57,488 (28.7%)	15,269 (31.58%)	2986 (34.3%)	
*Alcohol consumption*							< 0.001
Non-drinker	26,684 (53.48%)	73,915 (50.29%)	1194,785 (50.91%)	95,061 (47.46%)	23,709 (49.04%)	4451 (51.13%)	
Mild drinker	18,884 (37.85%)	60,898 (41.43%)	990,046 (42.18%)	86,606 (43.24%)	19,510 (40.36%)	3308 (38%)	
Heavy drinker	4323 (8.66%)	12,165 (8.28%)	162,125 (6.91%)	18,612 (9.29%)	5125 (10.6%)	946 (10.87%)	
*Regular exercise*	11,273 (22.6%)	31,627 (21.52%)	475,421 (20.26%)	40,687 (20.32%)	9720 (20.11%)	1570 (18.04%)	< 0.001
*Lowest quintile of income*	9949 (19.94%)	25,968 (17.67%)	386,574 (16.47%)	36,273 (18.11%)	9369 (19.38%)	1920 (22.06%)	< 0.001
*Diabetes mellitus*	29,645 (59.42%)	24,587 (16.73%)	106,737 (4.55%)	47,764 (23.85%)	41,688 (86.23%)	8703 (99.98%)	< 0.001
*Diabetes mellitus groups*							< 0.001
Non-diabetic	15,869 (31.81%)	109,721 (74.65%)	1,736,682 (74%)	40,374 (20.16%)	152 (0.31%)	0 (0%)	
Impaired fasting glucose	4377 (8.77%)	12,670 (8.62%)	503,537 (21.45%)	112,141 (55.99%)	6504 (13.45%)	2 (0.02%)	
New onset diabetes	1757 (3.52%)	1729 (1.18%)	23,521 (1%)	23,545 (11.76%)	21,667 (44.82%)	3066 (35.22%)	
Diabetes for < 5 years	11,444 (22.94%)	10,179 (6.93%)	40,812 (1.74%)	10,740 (5.36%)	7691 (15.91%)	2387 (27.42%)	
Diabetes for ≥ 5 years	16,444 (32.96%)	12,679 (8.63%)	42,404 (1.81%)	13,479 (6.73%)	12,330 (25.5%)	3250 (37.33%)	
*Fasting blood glucose in 2011 (mg/dL)*	110.69 ± 36.65	90.66 ± 22.45	94.11 ± 13.94	115.39 ± 22.35	163.57 ± 40.76	270.11 ± 69.12	< 0.001
*Fasting blood glucose in 2009 (mg/dL)*	185.72 ± 63.06	117.85 ± 23.62	93.9 ± 14.07	89.24 ± 21.17	106.78 ± 34.93	122.13 ± 40.54	< 0.001
*Hypertension*	24,976 (50.06%)	51,491 (35.03%)	610,929 (26.03%)	70,915 (35.41%)	23,457 (48.52%)	4484 (51.51%)	< 0.001
*Hypertension groups*							< 0.001
Non-hypertensive	9409 (18.86%)	41,341 (28.13%)	815,906 (34.76%)	49,551 (24.74%)	8175 (16.91%)	1335 (15.34%)	
Pre-hypertension	15,506 (31.08%)	54,146 (36.84%)	920,121 (39.2%)	79,813 (39.85%)	16,712 (34.57%)	2886 (33.15%)	
Hypertension	3649 (7.31%)	10,996 (7.48%)	164,396 (7%)	18,149 (9.06%)	4716 (9.76%)	876 (10.06%)	
Hypertension with medication	21,327 (42.75%)	40,495 (27.55%)	446,533 (19.03%)	52,766 (26.35%)	18,741 (38.77%)	3608 (41.45%)	
*Systolic blood pressure (mmHg)*	126.76 ± 15.28	123.94 ± 14.8	121.97 ± 14.46	125.26 ± 14.76	127.99 ± 15.18	128.51 ± 15.73	< 0.001
*Diastolic blood pressure (mmHg)*	77.86 ± 9.84	76.9 ± 9.79	76.08 ± 9.76	77.96 ± 9.86	79.07 ± 9.99	79.49 ± 10.36	< 0.001
*Dyslipidemia*	18,205 (36.49%)	34,415 (23.42%)	448,814 (19.12%)	51,641 (25.78%)	17,728 (36.67%)	3818 (43.86%)	< 0.001
*Dyslipidemia groups*							< 0.001
Total cholesterol < 240 (mg/dL)	31,686 (63.51%)	112,563 (76.58%)	1,898,142 (80.88%)	148,638 (74.22%)	30,616 (63.33%)	4887 (56.14%)	
Total cholesterol ≥ 240	2831 (5.67%)	10,001 (6.8%)	185,516 (7.9%)	20,072 (10.02%)	5075 (10.5%)	1065 (12.23%)	
Total cholesterol ≥ 240 with medication	15,374 (30.82%)	24,414 (16.61%)	263,298 (11.22%)	31,569 (15.76%)	12,653 (26.17%)	2753 (31.63%)	
*Total cholesterol (mg/dL)*	186.8 ± 38.65	191.4 ± 36.88	195.04 ± 35.92	198.88 ± 38.3	199.3 ± 41.97	203.47 ± 50.44	< 0.001
*High-density lipoprotein (mg/dL)*	51.34 ± 17.87	54.24 ± 22.2	55.41 ± 19.69	54.67 ± 20.29	52.2 ± 22.52	50.79 ± 19.77	< 0.001
*Low-density lipoprotein (mg/dL)*	106.12 ± 41.82	111.12 ± 42.39	114.95 ± 44.38	115.29 ± 42.59	112.33 ± 44.57	111.94 ± 47.56	< 0.001
*Chronic kidney disease*	4655 (9.33%)	8644 (5.88%)	115,903 (4.94%)	12,615 (6.3%)	4,330 (8.96%)	974 (11.19%)	< 0.001
*eGFR (mL/min/1.73m*^*2*^*)*	88.47 ± 40.44	90.95 ± 42.2	89.48 ± 36.92	87.82 ± 35.37	87.32 ± 39.63	86.46 ± 41.48	< 0.001
*Atrial fibrillation*	1298 (2.6%)	2970 (2.02%)	36,286 (1.55%)	3663 (1.83%)	1190 (2.46%)	222 (2.55%)	< 0.001
*Heart failure*	2438 (4.89%)	4663 (3.17%)	52,172 (2.22%)	5892 (2.94%)	2114 (4.37%)	434 (4.99%)	< 0.001

eGFR, estimated glomerular filtration rate; FBG, fasting blood glucose

### Temporal changes in FBG

**Fig. 2:**
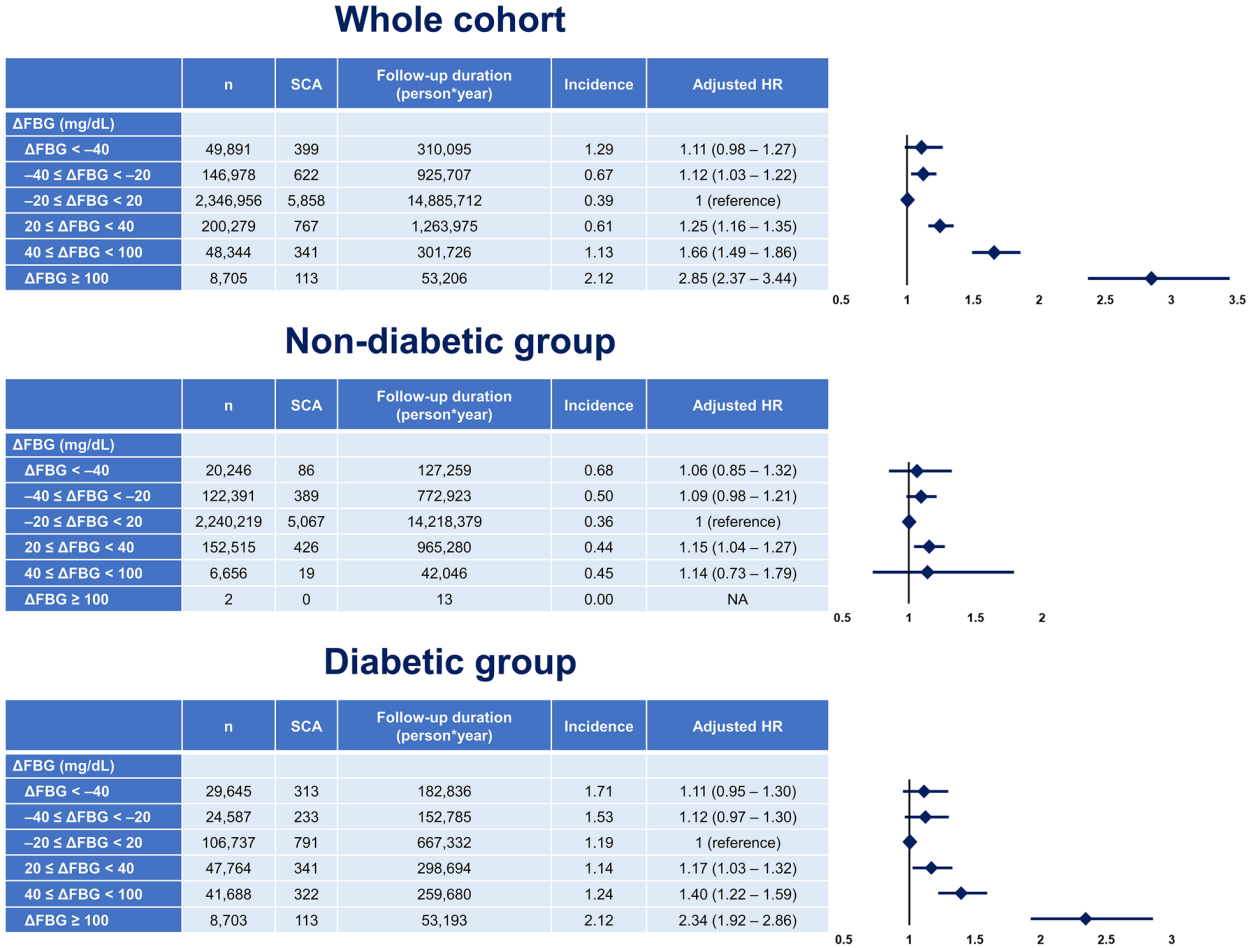
Risk of SCA according to ∆FBG. A significant association between ∆FBG and SCA risk was present in the whole cohort. That association was mainly observed in the DM group. People without DM did not show a clinically significant association between ∆FBG and SCA. DM, diabetes mellitus; FBG, fasting blood glucose; SCA, sudden cardiac arrest. Hazard ratios were adjusted for age, sex, body mass index, smoking status, alcohol consumption, regular physical activity, income level, hypertension, dyslipidemia, chronic kidney disease, heart failure, baseline FBG (measured in 2009), and DM duration (for diabetic group only; new-onset DM, DM for < 5 years, and DM for ≥ 5 years)

The incidence of SCA was lowest in people who maintained their ∆FBG within 20 mg/dL (euglycemic group; incidence = 0.39; Fig. 2 and Table 3). The highest incidence of SCA was observed in the ∆FBG > 100 group (incidence = 2.12; Fig. 2 and Table 3), and the ∆FBG < – 40 group also showed a higher incidence of SCA (incidence = 1.29; Fig. 2 and Table 3) than the euglycemic group. In the non-adjusted model, the − 20 ≤ ∆FBG < 20 group showed the lowest risk of SCA, and the ∆FBG < − 40 group (HR = 3.27; 95% CI 2.96–3.62; p < 0.001; Table 3) and ∆FBG > 100 group (HR = 5.41; 95% CI 4.49–6.52; p < 0.001; Table 3) had the highest risk of SCA (Additional file 1: Table S1).

**Table 3:** Risk of SCA according to ΔFBG

	n	SCA	Follow-up duration (person*years)	Incidence	Hazard ratio with 95% confidence interval			
					Univariate	Multivariate model 1	Multivariate model 2	Multivariate model 3
*ΔFBG (mg/dL)*								
ΔFBG < − 40	49,891	399	310,095	1.29	3.27 (2.96–3.62)	1.15 (1.01–1.31)	1.11 (0.98–1.27)	1.11 (0.97–1.26)
− 40 ≤ ΔFBG < − 20	146,978	622	925,707	0.67	1.71 (1.57–1.86)	1.16 (1.06–1.26)	1.12 (1.03–1.22)	1.12 (1.03–1.22)
− 20 ≤ ΔFBG < 20	2,346,956	5858	14,885,712	0.39	1 (reference)	1 (reference)	1 (reference)	1 (reference)
20 ≤ ΔFBG < 40	200,279	767	1,263,975	0.61	1.54 (1.43–1.66)	1.28 (1.18–1.38)	1.25 (1.16–1.35)	1.25 (1.16–1.34)
40 ≤ ΔFBG < 100	48,344	341	301,726	1.13	2.87 (2.57–3.20)	1.75 (1.56–1.95)	1.66 (1.49–1.86)	1.66 (1.49–1.85)
ΔFBG ≥ 100	8705	113	53,206	2.12	5.41 (4.49–6.52)	3.04 (2.52–3.67)	2.85 (2.37–3.44)	2.83 (2.35–3.42)

Incidence is per 1000 person*years of follow-up

FBG: fasting blood glucose; SCA: sudden cardiac arrest

Multivariate model 1: adjusted for age, sex, body mass index, smoking status, alcohol consumption, regular physical activity, income level, baseline FBG (measured in 2009)

Multivariate model 2: model 1 + hypertension, dyslipidemia, chronic kidney disease, and heart failure

Multivariate model 3: model 2 + coronary artery disease and ischemic stroke

**Fig. 3:**
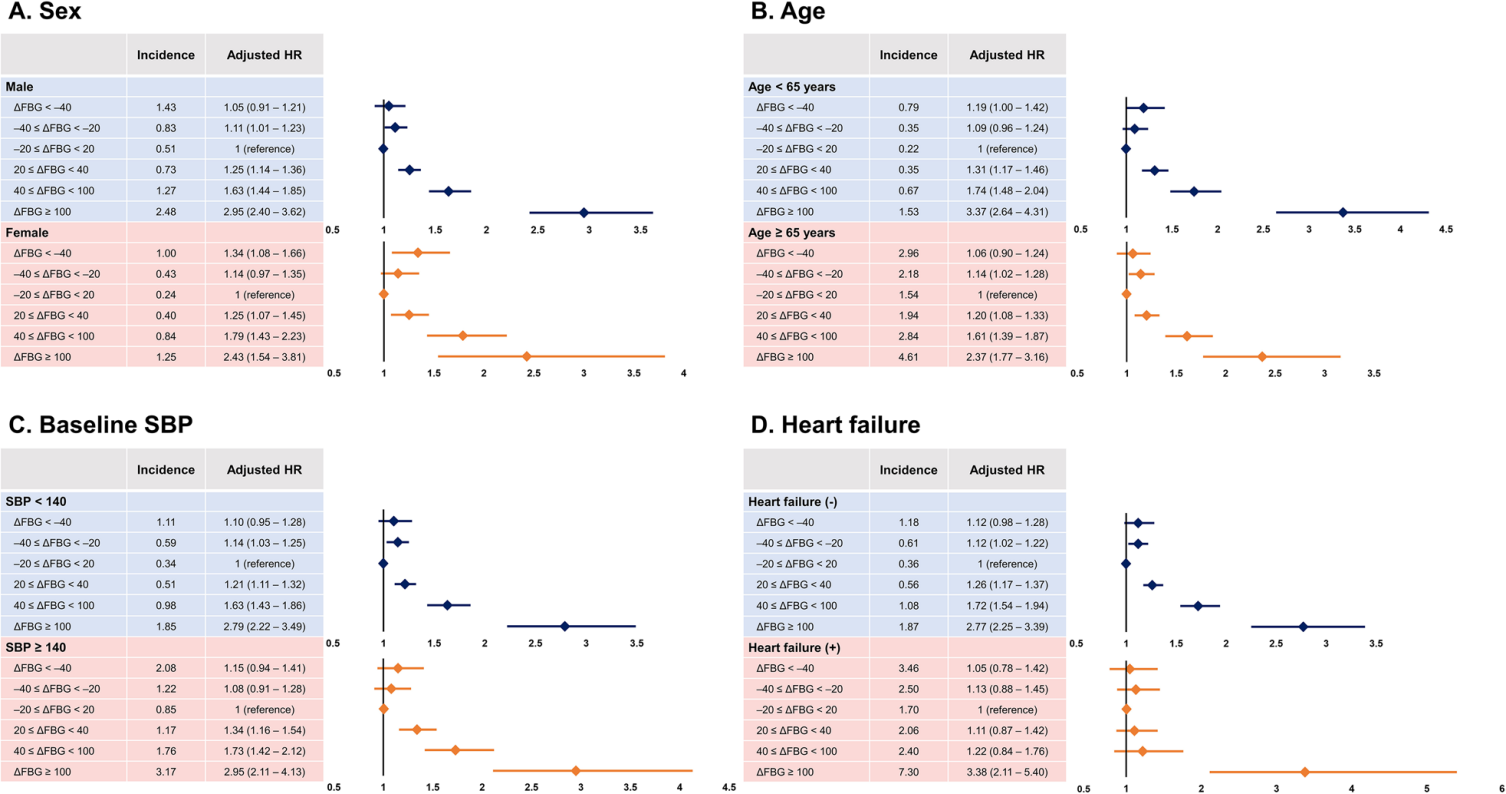
Subgroup analyses. The risk of SCA was associated with ∆FGB regardless of sex (**A**), age (**B**), baseline SBP (**C**), and the presence of heart failure (**D**). FBG, fasting blood glucose; SBP, systolic blood pressure; SCA, sudden cardiac arrest. Hazard ratios were adjusted for age, sex, body mass index, smoking status, alcohol consumption, regular physical activity, income level, hypertension, dyslipidemia, chronic kidney disease, heart failure, and baseline FBG (measured in 2009)

Baseline FBG (measured in 2009) varied significantly with ∆FBG and was adjusted in the multivariate model, in addition to age, sex, BMI, income, smoking status, alcohol consumption status, regular physical activity, hypertension, dyslipidemia, CKD, and heart failure. In the multivariate analysis, people who had more than a 100 mg/dL increase in FBG had a 2.85-fold higher risk of SCA than the euglycemic group (adjusted HR = 2.85; 95% CI 2.37–3.44; p < 0.001; Fig. 2). The risk of SCA was also increased in the 40 ≤ ∆FBG < 100 (adjusted HR = 1.66; 95% CI 1.49–1.86; p < 0.001; Fig. 2) and 20 ≤ ∆FBG < 40 (adjusted HR = 1.25; 95% CI 1.16–1.35; p < 0.001; Fig. 2) groups. Interestingly, the risk of SCA was increased in people with − 40 ≤ ∆FBG < − 20 (adjusted HR = 1.12; 95% CI 1.03–1.22; p = 0.009; Fig. 2), and people with ∆FBG < − 40 had a numerically increased risk of SCA without statistical significance (adjusted HR = 1.11; 95% CI 0.98–1.27; p = 0.113; Fig. 2). The association between ∆FBG and SCA risk was maintained when we considered only people with DM. However, in people without DM, there was no clear association (Fig. 2). The association between ∆FBG and SCA risk was maintained regardless of sex, age, baseline SBP, and the presence of heart failure (Fig. 3). In multivariate cox-regression model, 1 mg/dL increase in ∆FBG was associated with a 0.5% increased risk of SCA (adjusted HR = 1.005; 95% CI 1.004–1.005; p < 0.001; Table 4). Other risk factors associated with SCA was age, male sex, smoking, absence of regular exercise, low income, hypertension, CKD, atrial fibrillation, heart failure, and baseline FBG (measured in 2009) (Table 4).

**Table 4:** Risk factors for SCA

	Adjusted HR (95% CI)	p value
*Age (per 1 year)*	1.078 (1.076–1.080)	< 0.001
*Sex (male)*	2.479 (2.328–2.640)	< 0.001
*Body-mass index (per 1 kg/m*^*2*^*)*	0.966 (0.959–0.974)	< 0.001
*Smoking*		< 0.001
Non-smoker	1 (reference)	
Ex-smoker	1.132 (1.061–1.207)	
Current-smoker	1.872 (1.759–1.992)	
*Alcohol*		< 0.001
Non-drinker	1 (reference)	
Mild-drinker	0.806 (0.764–0.850)	
Heavy-drinker	0.898 (0.850–0.948)	
*Regular exercise*	0.898 (0.850–0.948)	< 0.001
*Income level (lowest quintile)*	1.065 (1.008–1.125)	0.026
*Hypertension*	1.434 (1.365–1.507)	< 0.001
*Dyslipidemia*	1.035 (0.983–1.089)	0.189
*Chronic kidney disease*	1.368 (1.285–1.457)	< 0.001
*Atrial fibrillation*	1.452 (1.317–1.600)	< 0.001
*Heart Failure*	1.652 (1.527–1.787)	< 0.001
*ΔFBG (per 1 mg/dL)*	1.005 (1.004–1.005)	< 0.001
*Baseline FBG (2009) (per 1 mg/dL)*	1.008 (1.007–1.009)	< 0.001

CI, confidence interval; FBG, fasting blood glucose; HR, hazard ratio; SCA, sudden cardiac arrest

## Discussion

The current study demonstrated that: (i) a long-term increase in FBG was significantly associated with increased risk of SCA; (ii) the degree of increase in ∆FBG was associated with the SCA risk, with the ∆FBG > 100 group having the highest risk; (iii) a long-term decrease in FBG showed no protective effect against SCA risk; and (iv) the association between ∆FBG and SCA risk was maintained in various subgroups. Despite the low incidence of SCA in our cohort, we were able to perform various subgroup analyses because of our large sample from a nationwide healthcare insurance database. Sequential measurements of FBG, long-term clinical follow-up, and no follow-up losses except through immigration are other strong points of our study.

### Strict FBG control

In our prior study, both DM and IFG were significantly associated with the risk of SCA [11]. In this study, ∆FBG was shown to be associated with the risk of SCA, indicating the importance of glucose homeostasis to prevent SCA. Fasting blood glucose is a traditional tool to diagnose DM and evaluate its severity. By obtaining sequential FBG measurements at a two-year interval, we were able to evaluate the association between glucose homeostasis and SCA risk. We found a significant association between baseline FBG (measured in 2009) and ∆FBG, so we adjusted for baseline FBG in the multivariate model to evaluate only the association between ∆FBG and SCA.

Diabetes mellitus is a known risk factor for atherosclerotic cardiovascular disease, including myocardial infarction and SCA [21, 22]. A prior study suggested that a higher HbA1c level might predict mortality from myocardial infarction or stroke [23]. That study is in accordance with the current study, which has demonstrated a significantly higher SCA risk in people whose FBG increases over time. The association between ∆FBG and SCA was obvious in people with DM but weak in people without DM. Our study thus provides epidemiologic evidence to emphasize the importance of glucose homeostasis in people with DM.

### Long-term decrease in FBG

We did not find any benefit of a decrease in FBG over time in terms of SCA prevention. The DIGAMI 2 trial demonstrated that intense glucose control in DM patients with myocardial infarction was not associated with any clinical benefit (death, reinfarction, or stroke) [24]. The intensive glucose control strategy in the ACCORD trial increased mortality during follow up and did not reduce the risk of major adverse cardiovascular events [25]. The ADVANCE trial also showed that intensive glucose control therapy did not reduce major macrovascular complications or death from any cause during follow up, despite its significant benefits on microvascular complications [26]. Our results are in accordance with those trials. Compared with people who maintained their ∆FBG within 20 mg/dL, people who had ∆FBG between − 40 and − 20 showed a 12% increased risk of SCA, which was statistically significant, and those who had more than a 40 mg/dL decrease in FBG showed a numerically increased risk of SCA (adjusted HR = 1.11; 95% CI 0.98–1.27). These results suggest that the adverse influence of hyperglycemia, such as that on the progression of atherosclerosis, might not be reversible by lowering serum glucose.

In contrast, several recent anti-diabetic drugs have been shown to reduce major adverse cardiovascular events. The EMPA-REG OUTCOME trial demonstrated that empagliflozin offered a considerable benefit in all-cause mortality during follow up (32% reduction) in DM patients [15]. In the LEADER trial, liraglutide also demonstrated a 15% reduction in all-cause mortality during follow up in DM patients [16]. Significant reduction in cardiovascular death, nonfatal myocardial infarction, or nonfatal stroke was observed with semaglutide [27]. Glucagon-like peptide-1 receptor agonist (such as liraglutide or semaglutide) was associated with significant mortality reduction in DM patients affected by coronavirus disease 2019 and anti-inflammatory and immunoregulatory effect was suggested as a potential mechanism which can have similar benefit in prevention of major adverse cardiovascular events [28]. However, dipeptidyl peptidase-4 inhibitors repeatedly failed to provide cardiovascular protection in DM patients [29–31]. Furthermore, dipeptidyl peptidase-4 inhibitors were found to increases vascular leakage in retina through VE-cadherin phosphorylation [32]. Furthermore, saxagliptin (one of dipeptidyl peptidase-4 inhibitors) was associated increased risk of heart failure admission in post-hoc analysis of SAVOR-TIMI 53 trial [33]. Therefore, we assume that various types of oral glucose lowering agents can have different impact on major adverse cardiovascular events and mortality in DM patients and that it might be the type of drug used for glycemic control that confers clinical benefit for cardiovascular outcomes, not the glucose reduction itself [15, 16, 27, 29–33].

## Limitations

This study has several limitations. First, our cohort consisted of only East Asians, so extrapolation of our results to other ethnic groups should be performed with caution. Second, the HbA1c level, a better marker of glucose control than FBG, was not available in this study. More frequent measurement of FBG might complement this limitation but FBG was only measured twice in this study. Lack of FBG data of the year of SCA occurrence is another limitation of this study. Third, coding errors might have occurred because our result is based on K-NHIS database and is a retrospective analysis. However, we have conducted various claims data-based analyses, and our coding strategy was validated in those studies [4, 17–20]. Various parameters (age, sex, medical history, medical measurement data, and social habits) were controlled in our multivariate model. Fourth, since the incidence of SCA is low, we enrolled people who underwent nationwide health screening in 2009 and 2011 instead of more recent years to have longer follow-up duration. We were not able to evaluate the impact of anti-diabetic medication since type of medication repeatedly changes during such a long follow-up duration.

## Conclusions

A long-term increase in FBG can be associated with increased risk of SCA. This association was independent of confounders and maintained among various subgroups. However, a long-term decrease in FBG was not associated with reduced risk of SCA. Whether maintenance of euglycemia or reduction in FBG can reduce the risk of SCA should be tested in future trials.

## Supplementary Information

**Additional file 1: Table S1.** Risk of SCA according to ΔFBG in various subgroups.

## Data Availability

No data are available. The ethical statement and the informed consent do not allow for free data availability.

## Abbreviations

BMI: Body mass index
CI: Confidence interval
CKD: Chronic kidney disease
DM: Diabetes mellitus
FBG: Fasting blood glucose
HR: Hazard ratio
ICD: International Classification of Disease
K-NHIS: Korean National Health Insurance Service
SBP: Systolic blood pressure
SCA: Sudden cardiac arrest
