# Supplementary material for: Long-term increase in fasting blood glucose is associated with increased risk of sudden cardiac arrest
Source: Cardiovasc Diabetol. 2023 Feb 20;22:38. doi: 10.1186/s12933-023-01764-0 (PMC9940683; doi:10.1186/s12933-023-01764-0)
Supplement: Supplementary file 1 — Additional file 1: Table S1. Risk of SCA according to ΔFBG in various subgroups. [file 12933_2023_1764_MOESM1_ESM.docx]

**Long-term Increase in Fasting Blood Glucose Is Associated with Increased Risk of Sudden Cardiac Arrest**

Yun Gi Kim,^1#^ Seung-Young Roh,^2#^ Joo Hee Jeong,^1^ Hyoung Seok Lee,^1^ Kyongjin Min,^1^ Yun Young Choi,^1^ Kyung-Do Han,^3^ Jaemin Shim,^1^ Jong-Il Choi,^1^* and Young-Hoon Kim^1^

^1^Division of Cardiology, Department of Internal Medicine, Korea University College of Medicine and Korea University Anam Hospital, Seoul, Republic of Korea

^2^Division of Cardiology, Department of Internal Medicine, Korea University College of Medicine and Korea University Guro Hospital, Seoul, Republic of Korea

^3^Department of Statistics and Actuarial Science, Soongsil University, Seoul, Republic of Korea

*Address for correspondence: Jong-Il Choi, MD, PhD, MHSc

^1^Division of Cardiology, Department of Internal Medicine, Korea University College of Medicine and Korea University Anam Hospital, Seoul, Republic of Korea

73 Goryeodae-ro, Seongbuk-gu, Seoul 02841, Republic of Korea

Tel: 82-2-920-5445 / Fax: 82-2-927-1478 / E-mail: [jongilchoi@korea.ac.kr](mailto:jongilchoi@korea.ac.kr)

^#^The first two authors contributed equally to this work.

**Running title:** Fasting blood glucose and sudden cardiac arrest

**Disclosure:** The authors have nothing to disclose.

**Total word count:** 6,942

**Table S1.** Risk of SCA according to ΔFBG in various subgroups.

|  | **N** | **SCA** | **Follow-up duration (person*years)** | **Incidence** | **Hazard ratio with 95% confidence interval** | | **p value for interaction** |
| --- | --- | --- | --- | --- | --- | --- | --- |
|  |  |  |  |  | **Univariate** | **Multivariate model** |  |
| **Male** |  |  |  |  |  |  | 0.363 |
| ΔFBG < –40 | 33,596 | 297 | 207,601 | 1.43 | 2.79 (2.48 – 3.14) | 1.05 (0.91 – 1.21) |  |
| –40 ≤ ΔFBG < –20 | 90,647 | 469 | 567,887 | 0.83 | 1.61 (1.46 – 1.77) | 1.11 (1.01 – 1.23) |  |
| –20 ≤ ΔFBG < 20 | 1,309,868 | 4,250 | 8,271,852 | 0.51 | 1 (reference) | 1 (reference) |  |
| 20 ≤ ΔFBG < 40 | 127,859 | 583 | 802,873 | 0.73 | 1.41 (1.30 – 1.54) | 1.25 (1.14 – 1.36) |  |
| 40 ≤ ΔFBG < 100 | 32,961 | 259 | 204,585 | 1.27 | 2.47 (2.18 – 2.80) | 1.63 (1.44 – 1.85) |  |
| ΔFBG ≥ 100 | 6,253 | 94 | 37,962 | 2.48 | 4.84 (3.94 – 5.94) | 2.95 (2.40 – 3.62) |  |
| **Female** |  |  |  |  |  |  |  |
| ΔFBG < –40 | 16,295 | 102 | 102,494 | 1.00 | 4.08 (3.34 – 4.99) | 1.34 (1.08 – 1.66) |  |
| –40 ≤ ΔFBG < –20 | 56,331 | 153 | 357,820 | 0.43 | 1.76 (1.49 – 2.07) | 1.14 (0.97 – 1.35) |  |
| –20 ≤ ΔFBG < 20 | 1,037,088 | 1,608 | 6,613,860 | 0.24 | 1 (reference) | 1 (reference) |  |
| 20 ≤ ΔFBG < 40 | 72,420 | 184 | 461,102 | 0.40 | 1.64 (1.41 – 1.91) | 1.25 (1.07 – 1.45) |  |
| 40 ≤ ΔFBG < 100 | 15,383 | 82 | 97,141 | 0.84 | 3.45 (2.77 – 4.31) | 1.79 (1.43 – 2.23) |  |
| ΔFBG ≥ 100 | 2,452 | 19 | 15,243 | 1.25 | 5.11 (3.25 – 8.03) | 2.43 (1.54 – 3.81) |  |
| **Age < 65 years** |  |  |  |  |  |  | 0.269 |
| ΔFBG < –40 | 37,926 | 188 | 238,873 | 0.79 | 3.53 (3.04 – 4.09) | 1.19 (1.00 – 1.42) |  |
| –40 ≤ ΔFBG < –20 | 120,297 | 268 | 762,970 | 0.35 | 1.57 (1.39 – 1.78) | 1.09 (0.96 – 1.24) |  |
| –20 ≤ ΔFBG < 20 | 2,036,964 | 2,895 | 12,958,383 | 0.22 | 1 (reference) | 1 (reference) |  |
| 20 ≤ ΔFBG < 40 | 166,664 | 367 | 1,057,646 | 0.35 | 1.55 (1.39 – 1.73) | 1.31 (1.17 – 1.46) |  |
| 40 ≤ ΔFBG < 100 | 37,680 | 159 | 237,648 | 0.67 | 3.00 (2.56 – 3.52) | 1.74 (1.48 – 2.04) |  |
| ΔFBG ≥ 100 | 6,907 | 66 | 43,014 | 1.53 | 6.89 (5.40 – 8.79) | 3.37 (2.64 – 4.31) |  |
| **Age ≥ 65 years** |  |  |  |  |  |  |  |
| ΔFBG < –40 | 11,965 | 211 | 71,222 | 2.96 | 1.94 (1.69 – 2.23) | 1.06 (0.90 – 1.24) |  |
| –40 ≤ ΔFBG < –20 | 26,681 | 354 | 162,738 | 2.18 | 1.42 (1.27 – 1.58) | 1.14 (1.02 – 1.28) |  |
| –20 ≤ ΔFBG < 20 | 309,992 | 2,963 | 1,927,329 | 1.54 | 1 (reference) | 1 (reference) |  |
| 20 ≤ ΔFBG < 40 | 33,615 | 400 | 206,329 | 1.94 | 1.26 (1.14 – 1.40) | 1.20 (1.08 – 1.33) |  |
| 40 ≤ ΔFBG < 100 | 10,664 | 182 | 64,078 | 2.84 | 1.85 (1.60 – 2.15) | 1.61 (1.39 – 1.87) |  |
| ΔFBG ≥ 100 | 1,798 | 47 | 10,192 | 4.61 | 3.03 (2.27 – 4.05) | 2.37 (1.77 – 3.16) |  |
| **Diabetes mellitus (-)** |  |  |  |  |  |  | 0.958 |
| ΔFBG < –40 | 20,246 | 86 | 127,259 | 0.68 | 1.90 (1.53 – 2.35) | 1.06 (0.85 – 1.32) |  |
| –40 ≤ ΔFBG < –20 | 122,391 | 389 | 772,923 | 0.50 | 1.41 (1.28 – 1.57) | 1.09 (0.98 – 1.21) |  |
| –20 ≤ ΔFBG < 20 | 2,240,219 | 5,067 | 14,218,379 | 0.36 | 1 (reference) | 1 (reference) |  |
| 20 ≤ ΔFBG < 40 | 152,515 | 426 | 965,280 | 0.44 | 1.24 (1.12 – 1.37) | 1.15 (1.04 – 1.27) |  |
| 40 ≤ ΔFBG < 100 | 6,656 | 19 | 42,046 | 0.45 | 1.27 (0.81 – 1.99) | 1.14 (0.73 – 1.79) |  |
| ΔFBG ≥ 100 | 2 | 0 | 13 | 0.00 | NA | NA |  |
| **Diabetes mellitus (+)** |  |  |  |  |  |  |  |
| ΔFBG < –40 | 29,645 | 313 | 182,836 | 1.71 | 1.45 (1.27 – 1.65) | 1.12 (0.96 – 1.31) |  |
| –40 ≤ ΔFBG < –20 | 24,587 | 233 | 152,785 | 1.53 | 1.29 (1.11 – 1.49) | 1.13 (0.98 – 1.31) |  |
| –20 ≤ ΔFBG < 20 | 106,737 | 791 | 667,332 | 1.19 | 1 (reference) | 1 (reference) |  |
| 20 ≤ ΔFBG < 40 | 47,764 | 341 | 298,694 | 1.14 | 0.96 (0.8 – 1.10) | 1.16 (1.02 – 1.32) |  |
| 40 ≤ ΔFBG < 100 | 41,688 | 322 | 259,680 | 1.24 | 1.05 (0.92 – 1.19) | 1.40 (1.23 – 1.60) |  |
| ΔFBG ≥ 100 | 8,703 | 113 | 53,193 | 2.12 | 1.80 (1.48 – 2.20) | 2.37 (1.94 – 2.89) |  |
| **Baseline SBP < 140 mmHg** |  |  |  |  |  |  | 0.839 |
| ΔFBG < –40 | 40,735 | 282 | 253,931 | 1.11 | 3.26 (2.89 – 3.68) | 1.10 (0.95 – 1.28) |  |
| –40 ≤ ΔFBG < –20 | 127,143 | 471 | 802,224 | 0.59 | 1.72 (1.57 – 1.89) | 1.14 (1.03 – 1.25) |  |
| –20 ≤ ΔFBG < 20 | 2,101,659 | 4,551 | 13,343,470 | 0.34 | 1 (reference) | 1 (reference) |  |
| 20 ≤ ΔFBG < 40 | 170,247 | 547 | 1,076,385 | 0.51 | 1.49 (1.36 – 1.63) | 1.21 (1.11 – 1.32) |  |
| 40 ≤ ΔFBG < 100 | 38,762 | 237 | 242,535 | 0.98 | 2.86 (2.51 – 3.26) | 1.63 (1.43 – 1.86) |  |
| ΔFBG ≥ 100 | 6,880 | 78 | 42,155 | 1.85 | 5.44 (4.35 – 6.80) | 2.79 (2.22 – 3.49) |  |
| **Baseline SBP ≥ 140 mmHg** |  |  |  |  |  |  |  |
| ΔFBG < –40 | 9,156 | 117 | 56,164 | 2.08 | 2.47 (2.04 – 2.98) | 1.15 (0.94 – 1.41) |  |
| –40 ≤ ΔFBG < –20 | 19,835 | 151 | 123,483 | 1.22 | 1.45 (1.22 – 1.71) | 1.08 (0.91 – 1.28) |  |
| –20 ≤ ΔFBG < 20 | 245,297 | 1,307 | 1,542,242 | 0.85 | 1 (reference) | 1 (reference) |  |
| 20 ≤ ΔFBG < 40 | 30,032 | 220 | 187,590 | 1.17 | 1.39 (1.20 – 1.60) | 1.34 (1.16 – 1.54) |  |
| 40 ≤ ΔFBG < 100 | 9,582 | 104 | 59,191 | 1.76 | 2.08 (1.70 – 2.54) | 1.73 (1.42 – 2.12) |  |
| ΔFBG ≥ 100 | 1,825 | 35 | 11,051 | 3.17 | 3.77 (2.70 – 5.28) | 2.95 (2.11 – 4.13) |  |
| **Atrial fibrillation (-)** |  |  |  |  |  |  | 0.691 |
| ΔFBG < –40 | 48,593 | 374 | 302,419 | 1.24 | 3.29 (2.96 – 3.65) | 1.12 (0.98 – 1.28) |  |
| –40 ≤ ΔFBG < –20 | 144,008 | 587 | 907,674 | 0.65 | 1.72 (1.58 – 1.87) | 1.14 (1.04 – 1.24) |  |
| –20 ≤ ΔFBG < 20 | 2,310,670 | 5,515 | 14,661,325 | 0.38 | 1 (reference) | 1 (reference) |  |
| 20 ≤ ΔFBG < 40 | 196,616 | 724 | 1,241,727 | 0.58 | 1.55 (1.43 – 1.67) | 1.26 (1.17 – 1.36) |  |
| 40 ≤ ΔFBG < 100 | 47,154 | 320 | 294,669 | 1.09 | 2.89 (2.58 – 3.23) | 1.68 (1.50 – 1.88) |  |
| ΔFBG ≥ 100 | 8,483 | 106 | 51,954 | 2.04 | 5.44 (4.49 – 6.59) | 2.88 (2.38 – 3.50) |  |
| **Atrial fibrillation (+)** |  |  |  |  |  |  |  |
| ΔFBG < –40 | 1,298 | 25 | 7,676 | 3.26 | 2.14 (1.43 – 3.21) | 0.95 (0.63 – 1.44) |  |
| –40 ≤ ΔFBG < –20 | 2,970 | 35 | 18,033 | 1.94 | 1.27 (0.90 – 1.80) | 0.88 (0.62 – 1.25) |  |
| –20 ≤ ΔFBG < 20 | 36,286 | 343 | 224,387 | 1.53 | 1 (reference) | 1 (reference) |  |
| 20 ≤ ΔFBG < 40 | 3,663 | 43 | 22,248 | 1.93 | 1.27 (0.92 – 1.74) | 1.09 (0.79 – 1.50) |  |
| 40 ≤ ΔFBG < 100 | 1,190 | 21 | 7,057 | 2.98 | 1.95 (1.26 – 3.03) | 1.47 (0.95 – 2.29) |  |
| ΔFBG ≥ 100 | 222 | 7 | 1,252 | 5.59 | 3.69 (1.75 – 7.80) | 2.39 (1.13 – 5.04) |  |
| **Heart failure (-)** |  |  |  |  |  |  | 0.440 |
| ΔFBG < –40 | 47,453 | 349 | 295,657 | 1.18 | 3.24 (2.91 – 3.61) | 1.12 (0.98 – 1.28) |  |
| –40 ≤ ΔFBG < –20 | 142,315 | 552 | 897,687 | 0.61 | 1.69 (1.55 – 1.84) | 1.12 (1.02 – 1.22) |  |
| –20 ≤ ΔFBG < 20 | 2,294,784 | 5,309 | 14,563,102 | 0.36 | 1 (reference) | 1 (reference) |  |
| 20 ≤ ΔFBG < 40 | 194,387 | 693 | 1,228,108 | 0.56 | 1.55 (1.43 – 1.68) | 1.26 (1.17 – 1.37) |  |
| 40 ≤ ΔFBG < 100 | 46,230 | 311 | 289,234 | 1.08 | 2.95 (2.63 – 3.31) | 1.72 (1.54 – 1.94) |  |
| ΔFBG ≥ 100 | 8,271 | 95 | 50,740 | 1.87 | 5.16 (4.22 – 6.32) | 2.77 (2.25 – 3.39) |  |
| **Heart failure (+)** |  |  |  |  |  |  |  |
| ΔFBG < –40 | 2,438 | 50 | 14,439 | 3.46 | 2.05 (1.53 – 2.74) | 1.05 (0.78 – 1.42) |  |
| –40 ≤ ΔFBG < –20 | 4,663 | 70 | 28,020 | 2.50 | 1.47 (1.15 – 1.89) | 1.13 (0.88 – 1.45) |  |
| –20 ≤ ΔFBG < 20 | 52,172 | 549 | 322,610 | 1.70 | 1 (reference) | 1 (reference) |  |
| 20 ≤ ΔFBG < 40 | 5,892 | 74 | 35,866 | 2.06 | 1.21 (0.95 – 1.55) | 1.11 (0.87 – 1.42) |  |
| 40 ≤ ΔFBG < 100 | 2,114 | 30 | 12,492 | 2.40 | 1.42 (0.98 – 2.05) | 1.22 (0.84 – 1.76) |  |
| ΔFBG ≥ 100 | 434 | 18 | 2,466 | 7.30 | 4.35 (2.72 – 6.95) | 3.38 (2.11 – 5.40) |  |

Incidence is per 1,000 person*years of follow-up.

FBG: fasting blood glucose; SBP: systolic blood pressure; SCA: sudden cardiac arrest.

Multivariate model: adjusted for age, sex, body mass index, smoking status, alcohol consumption, regular physical activity, income level, baseline FBG (measured in 2009), hypertension, dyslipidemia, chronic kidney disease, and heart failure.
